# Manpower capacity and reasons for staff shortage in primary health care maternity centres in Nigeria: a mixed-methods study

**DOI:** 10.1186/s12913-018-3819-x

**Published:** 2019-01-07

**Authors:** Joel O. Aluko, Rhoda Anthea, R. R. Marie Modeste

**Affiliations:** 1 School of Nursing, Faculty of Community and Health Sciences, University of the Western Cape, Bellville, Cape Town, South Africa; 2 Department of Physiotherapy, Faculty of Community and Health Sciences, University of the Western Cape, Bellville, Cape Town, South Africa; 3 Department of Nursing Sciences, Faculty of Health and Wellness, Cape Peninsula University of Technology, Bellville, South Africa

**Keywords:** Manpower, Staff shortage, Capacity, Primary health care, Maternity care, Nigeria

## Abstract

**Background:**

The heart-breaking maternal and neonatal health indicators in Nigeria are not improving despite previous interventions, such as ‘Health for all’ and ‘Millennium Development Goals. The unattained health-related goals/targets of previous interventions put the success of the new Sustainable Development Goals in doubt if the existing paradigm remains unchanged. Thus, mere branding of health policies without improving what constitutes the health system such as manpower capacity and quality as well as staff-patients ratio will be wasteful efforts. This issue of global public health concern provided an indication for describing the capacity of manpower and reasons for staff shortage in primary level of health that are providing maternity services to women and their new-borns in Nigeria.

**Methods:**

This is an embedded mixed-methods study. Its quantitative strand collected data with the aid of a structured questionnaire from 127 health workers across the 21 purposively selected primary health care centres in five local government areas. Descriptive statistics were employed for analysis. The qualitative strand of the study collected data through in depth interviews from medical officers of health or their representatives. The tape recorded and transcribed data were thematically coded, while reporting was by direct quotes. The mixing of the data from both strands was done in the discussion section.

**Results:**

Twenty-nine (22.8%) of the health workers were between ages 51–58; 111 (87.4%) were married, while 44 (34.6%) had worked for duration of 21–33 years in service. Evidences of incompetence were observed among the health workers. A total of 92 (72.4%) had been performing episiotomies on women in labour. Similarly, 69.8% had been repairing vaginal traumas. Nine (7.1%) knew the necessary steps of controlling postpartum vaginal bleeding, while 115 (91.3%) of them had not been trained in Life-Saving Scheme and post-abortions care.

**Conclusion:**

The shortage of manpower, disproportional skilled/semi-skilled ratio, lack of framework for staff recruitment, staff incompetence and inappropriate childbirth practices show that women were not receiving quality maternal and neonatal cares at the maternity centres.

## Background

Globally, more than one billion people (women and children being the majority) lack access to quality health services [1]. The huge shortage, imbalanced skill mix and uneven geographical distribution of health workers in health services, including maternity service have been acknowledged in most countries [2]. The high maternal and neonatal death rates recorded in Nigeria are unacceptable and require urgent and permanent solutions [3]. Severe shortage of qualified healthcare workers, lack of political will to recruit appropriate categories of health workers to practice within the scope of their respective training and inaccessibility of skilled care to many women are issues of serious concern to the researchers [4]. The effectiveness of maternity care services depends largely on the number and the quality of human resources available to respond to the emerging health needs of women and their newborns.

The perennial shortage and queried competence of manpower in maternity care providing institutions is capable of impacting negatively on quality of services rendered to women and their newborns. The possible consequence of compromised quality of maternity services described above have a far-reaching influence on the outcome of pregnancy. This is because poor quality of maternity service presents itself in the form of sub-standard care provision, unnecessary interventions, disrespectful treatment of women, denial of women’s choice of birthing positions and disallowing significant others to stay with women in labour [5]. In addition, subsequent maternal and neonatal complications that may necessitate referral to higher levels of health care facilities are often delayed or denied [5]. Quality maternity care is the degree to which maternity services for women and their newborn populations increase the likelihood of timely and appropriate treatment for the purpose of achieving desired outcomes and are consistent with current professional knowledge and uphold basic reproductive rights [1].

In order to combat the maternal and neonatal deaths that have been sustained close to a decade, manpower populations, health workers competence and staff recruitments as factors of quality maternity care ought to lie at the core of all strategies for accelerating progress towards Sustainable Development Goal (SDG) three (3), targets 1 and 2. The SDG 3 aims at reducing “the global maternal mortality ratio to less than 70 per 100,000 live births by 2030”; and “ending preventable deaths of new-borns and children under five years of age, with all countries aiming to reduce neonatal mortality to at least as low as 12 per 1,000 live births and under-five mortality to at least as low as 25 per 1,000 live births by 2030” [6]. The reasons for analysing the situation of manpower and staff recruitment for maternity care provision at the primary health care level are many. First, most users are from low socio-economic backgrounds and often find it difficult to afford the cost of services at a higher level of care. Second, this category of women is more than their counterparts who could access and afford a higher level of care without constraints [6, 7]. Third, this category of nation’s population rarely has any form of health insurance [7]. Fourth, the accessibility of these women to formal health care facilities is frequently influenced by culture, ethnicity and economic status. Fifth, they are frequently confronted with the problem of shortage of health workers to meet their health care needs [7]. Lastly, they belong to the vulnerable group who are likely to face challenges of getting the right care at the right time [7]. In addition; there is a serious dearth of literature regarding human resource status, capacity and competence at the primary level of care in Nigeria. The reason for the dearth was because most studies focused on higher level of health facilities. All the above cumulatively form the premise for this current study.

## Methods

### Study design

This study utilized embedded mixed methods research design to analyse the situation of manpower and staff recruitment for primary health care-based maternity centres in five local government areas of a city in South-West Nigeria. There are four types of mixed methods research designs namely: the exploratory, the explanatory, the triangulation and embedded mixed methods designs. The embedded mixed methods research design is different from the other three [8, 9] Unlike the quantitative and the qualitative research designs, mixed methods research designs have unique advantages Mixed methods research designs enable investigators to address a range of confirmatory and exploratory questions simultaneously [8, 9]. The designs give opportunity for a greater assortment of divergent views and make them alert to the possibility, that issues are more multifaceted than they may have initially supposed [8, 9] The embedded mixed methods design was chosen for this study, so that the qualitative strand could provide a supportive secondary role to the primary quantitative strand of the study, because a single quantitative dataset was considered insufficient to elicit all necessary information requires for the study [8].

### Study settings

This study was done in the five local government areas (LGAs) of one of the urban cities in South-Western Nigeria. The city became popular as a result of the three major factors. One, it is a state capital city. Two, it houses primary, secondary and tertiary level health institutions. Three, the city is densely populated with an estimated population of about two million; most of the population live in slums and high-density areas [7].

### Instruments and data collection

The study utilized both quantitative (a structured questionnaire) and qualitative (in depth interviews) research tools for data collection regarding the facilities and the health workers. Quantitatively, the information elicited includes socio-demographic variables, competency and labour management practices of the health workers. In addition, the participants were required to list maximum of four components of safe motherhood initiative. Each component carried one point. Thus, the maximum obtainable score was 4. Similarly, the health workers were required to state the steps they usually took when helping women who were bleeding after childbirth. A maximum of six steps were required, namely: assessment of the perineum/vagina, palpation of the uterus, removal of blood clots in case of uterine atony, setting of intravenous infusion line, parenteral administration of oxytocic drugs and send for a doctor/referral to higher level of care. The responses of the participants were scored, with 6 points being the maximum obtainable score. In the rating, participants were categorized thus: 0 = No idea, 1–2 = some idea, 3–6 = Good idea. Subsequently, the level of their competencies was assessed against the categories of the health workers.

Furthermore, the qualitative strand of this study was done among two distinct populations, namely: Medical Officers of Health (MOHs) and the Head of facilities. The qualitative aspect employed in-depth interview (IDI) to elicit information from the MOHs and heads of facilities.

The face and content validities of the questionnaire were ensured by comparing its items with previous studies and by matching them with the stated objectives and formulated research hypotheses. In order to ensure reliability of the instrument, the instrument was pilot-tested in a LGA that was homogeneous to the selected five LGAs. The reliability coefficient of the survey instrument (questionnaire) was 0.8. In addition, ethical clearances were obtained from University of the Western Cape, South Africa and Oyo State Ethical Committee. All relevant principles were strictly adhered to.

### Quantitative strand - sample size and sampling techniques

The 130 sample size was arrived at based on Stoker’s sample size determination table, which represented 20% of 540 of the health workers [10]. The study utilized a multistage sampling technique: The five LGAs were selected purposively; based on proximity to the researcher. Also, the 21 PHC-based maternity centres across the five LGAs were selected purposively. Thus, only PHC centres that were designed to provide maternity services were studied purposively. A systematic random sampling technique was utilized to recruit **130** health workers from estimated populations of **540.** The duty rosters served as sample frames for the health workers. The sample intervals were calculated for the population using the statistical formula: K = N/n. Where K = Sample interval; N = Total population in the sample frame; n = sample size. Thus, the sample interval was 4. The starting point for sample selection was determined using simple random (balloting) and health workers with serial number 2 on the sample frame (duty rosters) were used as starting point in all the PHC centres.

#### Inclusion criteria

These included all categories of caregivers, such as nurse/midwives, community health officers (CHOs), community health extension workers (CHEWS) and health assistants (HAs) who regularly performed physical examinations on antenatal women and attended to women during labour and childbirth.

#### Exclusion criteria

Health workers who performed just supportive roles (such as pharmacy technicians, medical record officers, laboratory technicians and cleaners) were excluded from the study.

### Qualitative strand - sample size and sampling techniques

The participants for the qualitative strand of the study were selected by non-probability sampling methods. All the 5 MOHs (1 from each LGA) together with 5 heads of facilities (1 from each LGA) were purposively selected for IDI sessions. However, one of the MOHs in one of the LGAs was not available and, therefore, he could not participate in the study.

## Results

### Quantitative aspects

Out of the 130 health workers recruited for the study, 127 were available for the study across 21 PHC facilities, where maternity services are being rendered. Thus, the response rate was 97.7%. The described components are adequacy of health workers, health workers’ competence, situation of manpower recruitments and exits (in the form of retirement, resignation, retrenchment or death).

#### Socio-demographic attributes of the participants

The mean age of the health workers who participated in the study was 41 years ±10 SD. Health workers within the age bracket 51–58 years were 29 (22.8%), while those who had spent between 21 and 33 years in service were 44 (34.6%) (Table 1).

**Table 1:** Socio-demographic variables of health workers (*N* = 127)

Socio-demographic variables	N	%
Age groups		
20–30 years	25	19.7
31–40 years	33	26.0
41–50 years	40	31.5
51–58 years	29	22.8
Duration in specialty		
1–10 years	43	33.9
11–20 years	40	31.5
21–33 years	44	34.6
Marital status		
Single	14	11.0
Married	111	87.4
Divorced	1	0.8
Widowed	1	0.8
Profession		
Health Assistants	16	12.6
CHEWs	58	45.7
CHOs	13	10.2
Nurses/Midwives	33	26.0
Laboratory Technicians And Record Officers	7	5.5

*Key: CHEWs* Community Health Extension Workers

*CHOs* Community Health Officers

#### Competence of health workers

Out of 127 health workers studied, 64.6% heard of the concept of ‘safe motherhood’ but only 10.2% had idea of its components. The participants’ score ranged between 0 and 3 points; their mean score ± standard deviation was 0.45 ± 0.7. The scores of the participants were further classified thus: 0 = No idea, 1 = little idea, 2–3 = good idea. Participants who had no idea about the concept were 66.9%, while those who had good idea were 8.7%. The remaining 24.4% had little idea about the concept.

**Fig. 1:**
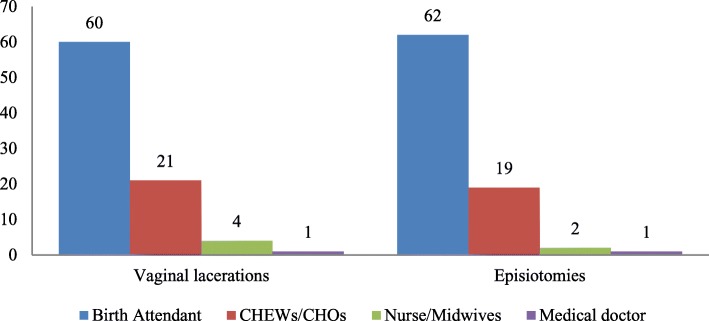
Categories of health workers who repaired vaginal lacerations and episiotomies in the PHC facilities

In addition, 76.4% of the health workers said that Focused Antenatal Care (FANC) was being practised in their respective Primary Health Care (PHC) facilities, but the remaining participants declared that orthodox antenatal care, otherwise known as traditional antenatal model, was still being practised. A total of 115 (90.6%) of them had been attending to women in labour, while 7 (5.5%) did not disclose whether or not they had been attending to women in labour and during child delivery in their facilities. Besides, 67.7% said that at least one woman had sustained vaginal laceration at one time or the other during conduct of child delivery. Out of 86 (67.7%) who had witnessed at least a case of vaginal laceration, 69.8% used to have repaired the vaginal trauma by themselves, while 30.2% had to wait for another health worker; either a Community Health Extension Worker (CHEW) or a Community Health Officer (CHO) or a nurse/midwife or a medical doctor for surgical repair of the vaginal trauma (Fig. 1). Similarly, a total of 92 (72.4%) of the health workers had performed deliberate surgical cutting on vaginal muscles (episiotomy) for women in labour, while the remaining had never attempted it on any woman. Out of these 92 health workers, 67.4% had attempted repair of the episiotomies that were performed on clients but the remaining had to wait for other health workers to assist them in carrying out the repair (Fig. 1). The outcomes of management of episiotomies were not assessed in this study.

The health workers were required to state the steps they usually took when helping women who were bleeding after childbirth. A maximum of six steps were required. Nine (7.1%) of the health workers were able to list six necessary steps (actions) that a birth attendant must take to help a woman who experienced bleeding after childbirth. Some of the health workers who were able to list certain actions that could be considered appropriate to arrest postpartum bleeding did not list in a sequential order. The responses of the participants were scored, with 6 points being the maximum obtainable score. In the rating, health workers were categorized thus: 0 = No idea, 1–2 = some idea, 3–6 = Good idea. A total of 63 (49.6%) had no idea of the necessary steps to take in order to help women who bleed after childbirth, while 30 (23.6%) and 34 (26.8%) had little and good ideas on the necessary steps to achieve arrest of bleeding following childbirth. The nurse/midwives demonstrated higher level of competence than other category of health workers (HAs, CHO, CHEWs etc.). This was found to be statistically significant; *p*-value being 0.001 (Table 2).

**Table 2:** Competencies of categories of health care workers in PHC facilities

Categories of health workers	Level of competence			Chi-sq.	df	*p*-value
	No idea	Little idea	Good idea			
Other health workers	52 (82.5%)	26 (86.7%)	16 (47.1%)	17.723	2	0.001
Nurse/Midwives	11 (17.5%)	4 (13.3%)	18 (52.9%)			

**Fig. 2:**
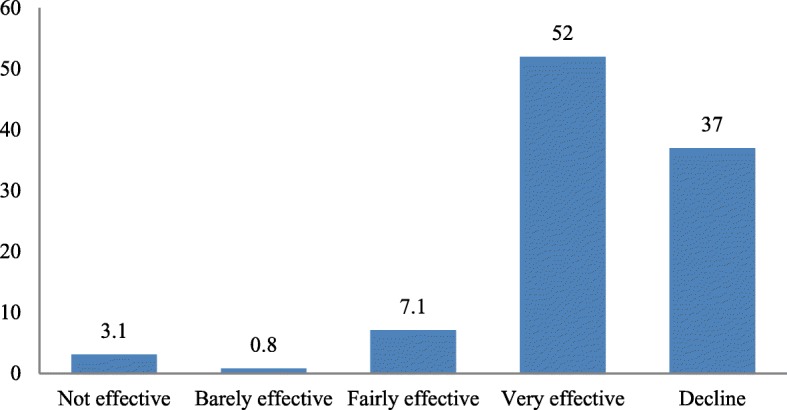
Participants’ views of effectiveness of prenatal care in their facilities

Out of the health workers, 52% affirmed that the prenatal care they were providing for women in their facilities was very effective but 40.2% affirmed that it was not effective. Figure 2 shows the various views of the health workers about the effectiveness of the prenatal care services rendered to women in the PHC facilities across the five local government areas.

**Fig. 3:**
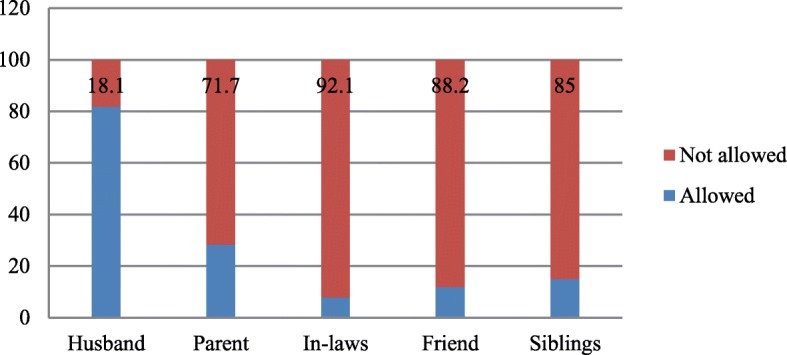
Categories of people health workers would allow into the delivery room

The health workers in the PHC facilities determined the categories of significant others that would be allowed into labour ward when attending to women in labour. A total of 81.9% of the health workers would allow husbands to stay with their wives in labour room. Figure 3 shows the categories of people the health workers would allow into the labour room during childbirth.

**Fig. 4:**
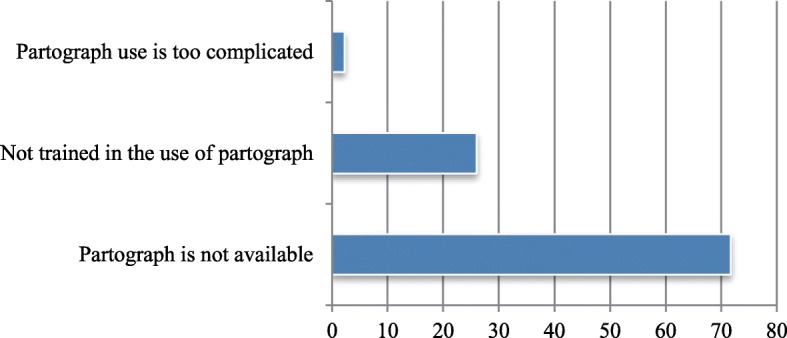
Factors responsible for non-use of partograph in the PHC facilities

None of the health workers used partograph for progress of labour monitoring in any of the health facilities studied. Various reasons were reported for non-use of partograph for monitoring the progress of labour in the health facilities. Unavailability of partograph sheet/form topped the list (71.7%) of the reasons reported in this study. Besides, 26.0% of the participants said they were not trained in the use of partograph for monitoring labour progress (Fig. 4). Moreover, 91.3% of the health workers had not been trained in Life-Saving Scheme (LSS) and post-abortions care (PAC). The eleven (8.7%) health workers who stated that they were trained in LSS and PAC could not mention at least one out of the components of LSS.

Various risky practices among the health workers while attending to women in labour and during child delivery process were recorded in this study. These risky practices include:

i. Applying pressure on the abdomen of the women in labour to facilitate quick delivery.
ii. Administration of intravenous oxytocic agent (syntocinon) while babies were still in uterus.
iii. Beating of women in labour to forcefully gain their co-operation.
iv. Shouting on and bullying women in labour to separate their thighs for vaginal examination and childbirth.

Table 3 shows the risky practices of health workers while attending to women in labour and during childbirth.

**Table 3:** Risky practices by health workers to speed up second stage of labour (*N* = 127)

Risky practices	Practiced n (%)	Not practiced n (%)
Applying abdominal pressure	42 (33.1)	85 (66.9)
Giving of IV oxytocin bolus	24 (18.9)	103 (81.1)
Asking client to bear down with or without full cervical dilatation	77 (60.6)	50 (39.4)
Beating client to gain her cooperation	12 (9.4)	115 (90.6)
Forcing client to keep her thighs separated	36 (28.3)	91 (71.7)
Gaining client’s cooperation by explanation	93 (73.2)	34 (26.8)
Inviting a relation to scold the client	53 (41.7)	74 (58.3)

**Fig. 5:**
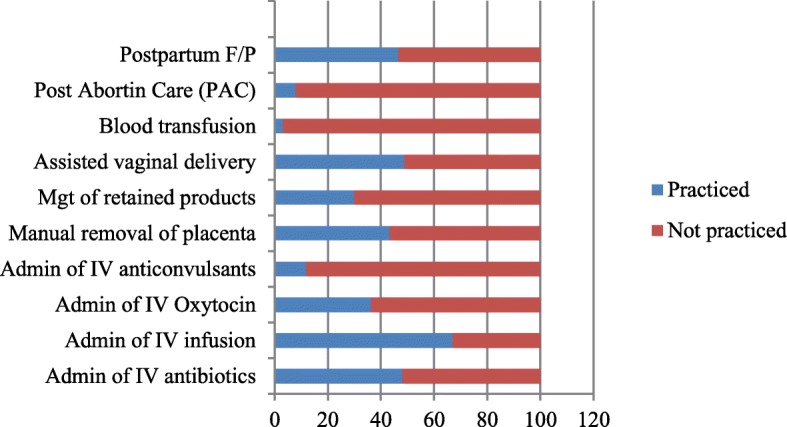
Procedures adopted by the health workers in the PHC facilities during postpartum haemorrhage

Among the health workers, 57.5% said that they could perform manual removal in cases of retained placenta, while 11.0% did not know what to do when a woman had retained placenta (Table 4). Seventy-two (56.7%) of the health workers had not been trained on how to perform manual removal of retained placenta or retained products of conception. Table 5 shows various actions, which the health workers usually undertook when postpartum bleeding occurred. Similarly, 18.9% of the health workers did not know what to do to help women with postpartum haemorrhage (bleeding). Figure 5 shows the services that health workers performed in the PHC facilities.

**Table 4:** Actions performed by health workers to help women with retained placenta

Actions performed by health workers	N	%
Manual removal	73	57.5
Send and wait for the doctor	7	5.5
Referral to a higher level of care	36	28.3
Don’t know what to do	11	8.7
Total	127	100.0

**Table 5:** Actions taken by the health workers to help women with postpartum bleeding

Health workers’ actions to mitigate postpartum bleeding	N	%
Packing of vagina with sanitary pad	47	37.0
Send and wait for the doctor	2	1.6
Referral to a higher level of care	36	28.3
Setting of IV infusion followed by referral	18	14.2
Don’t know what to do	24	18.9
Total	127	100.0

**Fig. 6:**
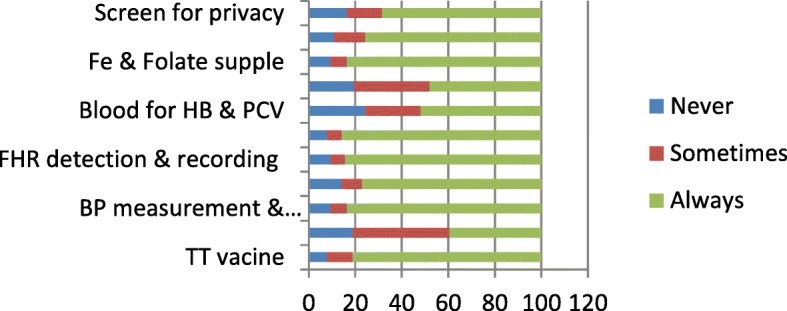
Actions taken on prenatal women during clinic visits in the facilities

The next figure (Fig. 6) reveals that diagnosis of sexually transmitted infections (STIs), blood samples for haemoglobin/packed cell volume and urine test for proteins and bacteriuria were given the least attention during antenatal clinic visits.

Although, none of the PHC facilities had postnatal care clinics as at the time of data collection, but clients with complaints were cared for whenever they reported to the facilities. Routine postnatal assessments were never done for women by the health workers. However, the care rendered to women who voluntarily reported and were attended to during the postnatal period was considered as postnatal service by 70.9% of the health workers. A total of 74.0% of the health workers attested to the fact that the serum bilirubin of babies that were born in their health facilities was not monitored.

#### Emerging themes in the quantitative strand of the study

Certain important themes emerged from the survey. They are gross shortage of manpower, disproportional skilled/semi-skilled health workers ratio, staff incompetence and inappropriate childbirth practices.

### Qualitative aspects

#### Health workers/client ratio

In this study, staff capacity refers to the population of staff working in the PHC centres in relation to the rate of client flow. In addition, staff capacity describes the ratio of qualified nurse-midwives population to that of other categories of health workers in the PHC centres. Therefore, staff capacity addresses all the parameters indicated in the timeliness, appropriateness and consistency of services rendered to clients.

In order to explore how one medical doctor can effectively cover all the PHC centres in his/her LGA, this question was posed to them: “How many of you have seen a medical doctor since you’ve been coming to this clinic?” In a form of non-verbal communication, one out of the twelve responded by raising hand. The staff/patient ratio in all the PHC facilities was described as one of the barriers to provision of quality maternity care to mothers and their newborns. The serious persistent shortage of health workers across all the PHC centres was mocked by a Chief Nursing Officer (CNO) during the interview. Her mocking statement reads thus:

“Ha...ha...ha...ha… (She laughed) you know normally, eh….medically, it should be one nurse to three clients or patients but that is not operating in the government. It may be a nurse ha-ha…ah (she laughed) to forty patients, ha...ha...ha...ha... (she laughed). The adequacy is not there..., there’s no way you will do the roster, and it’s just only one person that you would put on afternoon. And that afternoon, minor ailments will come, those on daily injections will come, then you may have labour cases. So, it is when these people (unskilled health workers) join us, despite the fact they are not paid, I just have to plead with them that they cannot be on morning shift only. That is when they agreed to work in the afternoon.”

#### Nurse-midwives/other health workers ratio

Furthermore, the responses from the interviews revealed that in all the facilities studied, the reported staff shortage affected the nurse/midwife category more than other categories of health workers - the CHEWs and the health Assistants (HAs). In other words, the CHEWs and the HAs were more than the nurses and nurse-midwives put together. Therefore, the CHEWs and the HAs were seen by clients/patients as nurses in those facilities. Most women could not differentiate other health workers from qualified nurse/midwives. The extreme shortage of nurse/midwives across all the facilities made the CHEWs and the HAs to perform the roles and the functions of midwives in the facilities – a situation that became inevitable. The scenario credited to a CNO that follows below lends support to the above assertion:

*“*You know, we don’t have a choice, in cases when you know the right thing to do but there is nothing to make use of, then you use what you have. ‘*Se’* (do) you understand? The conduct of child delivery is not part of their curriculum, they learn on duty as apprentices*.”*

She explained further why the situation had persisted:

“In some centres, they have just one nurse, and one nurse cannot be on morning, afternoon and night; it is not possible …. So, you make use of what you have.”

#### Staff recruitment and exit

The interviews also showed that the problem of shortage of health workers, which cut across all the LGAs in Oyo State, Nigeria, was due to lack of regular staff recruitment in each of the LGAs. The quote below credited to a medical officer of health (MOH) supports the above statement:

“Yes, yes, you are quite right... but it is not limited to this local government area.... That is the picture in all the LGAs in the state. And the reason being that, there is em...embargo on employment at present by the state government. And still some people are getting out of service year in year out and they are not being replaced....”

In addition, why nurses/midwives were far less than other categories of health workers was linked to lack of regular recruitment exercises despite ongoing retirement, resignation, deformities or death of staff at one particular time or the other. A medical doctor in one of the LGAs painted the described situation thus:

“I wouldn’t know why that has been so, em... as I said here earlier, since I joined service, em... over 15 years ago, they’ve not recruited em... new nurses since that time. It’s not as if em... they recruited other cadres of health staff but more people like health assistants, they are climbing the ladder... So, they prefer, maybe because of their qualifications, their school certificate or something that they have... might not be good enough for them to enter school of nursing. So, most of these lower cadre staff, they go to school of hygiene to... for... em... for... health extension worker courses. So that’s why you find more of them around, em..., more of the CHEWs and CHOs cadre around. But, since they have not really recruited, I wouldn’t know what it was before we joined the service. But, when we joined the service, the ratio of nurses to other cadres has always been too low.”

When she was probed further on whether or not she realized the effects of the unfavourable state of manpower in the LGA where she served, she replied in the affirmative thus:

“Yeah! Yeah! It can affect and we, we, we have been advocating to... for them to be recruited... We have been getting good response, because recently a letter was sent from the Local Government Service Commission. They want to know the number of nurses, though other cadres were included too... May be they are planning to recruit more, I don’t know, but I feel it’s a, it’s a right decision....”

During the in-depth interview (IDI) sessions, the shortage of manpower, which had become a usual phenomenon in all primary level of health care in all the five LGAs, was attributed to lack of regular recruitment exercises. In one of the LGAs, a MOH narrated her experience since she joined the service of the LGA many years back. She explained further that such situation was beyond the level of a career officer like her. Her statement is quoted below:

“Well... em... so many things are responsible. One, em... I can’t remember the last time any recruitment was made at this level and em... people retire every now and then. This year, I have about three or four of members of my staff that retired and there have been no replacement for them and even besides that, people are being redeployed to other local governments because of the mass posting that took place late last year. Some people felt this place is too far from their place of residence. So, they have been clamouring for reposting and some of them were posted to other local governments. So, so many factors are responsible but, what do you do? It’s beyond ... though we have been pressing them (the government), we are pressurizing them (the government) to get us more staff. We are hopeful that em... they would respond to our call*.”*

#### Explanation of staff/client ratio disproportion across PHC facilities

The findings of this study showed that no LGA has more than one medical doctor who occupied the position of the Medical Officer of Health (MOH) in his/her respective LGA. The MOH combined clinical consultations and administrative functions, the latter being more dominant than the former. However, all the MOHs concerned viewed the situation as inadequate when compared with the different levels of facilities and numbers of client population each was to serve. For example, when one of the medical doctors was asked about the number of doctors available in his local government area, she replied: “Just one.” In order to confirm the answer she gave, she was quizzed further: Are you the only one? She replied affirmatively, “Yes!” Her response surprised the researcher and he probed further: “Don’t you think you need other medical doctors to assist you?” To this question she replied thus:

“...we need! If the Local Government Service Commission is willing to recruit more doctors... Okay? So, if they are willing to recruit more. There’s nothing em..., there’s nothing bad in having two, three doctors in the Local Government. It will make the job em...easier.”

The same medical doctor’s further explanation on the issue of staffing goes thus:

“Now, when you look at the population of a local government of about 400,000 people; this LG happened be one of the most populous, the densest LGA in Ibadan and ditto for Oyo State or let me just say Oyo State as a whole. Look at that ratio... one doctor... you understand? I understand there are private and other public health facilities. People come from all over..., from not necessarily from... this LGA em... so, prorating it... generally, there is...you know... a wide gap...you know..., in human resource and it is a big challenge. You can almost say a doctor to about 100,000 populations. So now, come to the other one, presently as it...em.. I have about 23 nurses right; I have em...20 CHOs, I have em...39 CHEWs, I have 6 JCHEWs, I have 22 HAs, I have em....3 medical laboratory personnel, 2 pharmacy technicians. So, when you look at this spread..., this spread...then, you know... to 14 facilities, How, how em...do you share that to large number of health facilities. So, manpower is really a serious challenge on our hand. I only, I only, I only want to see this challenge improved upon.

She added that:

In type 3 facilities...once they offer 24 hour service at least minimum of 5 nurses are to be in that level of facility. Minimum! Minimum! To run three shifts: morning, afternoon, night. Now, if we are saying 23 nurse/midwives..., now, 6 of my facilities fall under type 3 facilities. All right! Now, when you multiply, you say, 5 times 6. That comes to about minimum of about 30 nurse/midwives. They are to be at that level alone...at that level.... That is not to say that the other ones, ha... ha...ha...ha...; the other ones must still have. I’m not even talking about the comprehensive that must have at least minimum of 8, because of the volume of the work there, Now, if we then saying in the ...local government as a whole we have 23 nurse/midwives. This will give you the insight that really will have a serious challenge, because the minimum of 5 in type III facility –that is the minimum package as far as human resources is concerned; going by the minimum requirements as stipulated by the National Primary Health Development Agency.”

Another important role of the LGA is funding. In this study the LGAs were identified as the major funder of PHC. However, other international agencies were said to be supporting certain aspects of PHC programmes. This report was captured from the response of a participant during one of the IDI sessions:

“...but... the local government funds it, though we...,we...,we...receive support from em...multilateral, bilateral agencies like UNICEF, WHO, USAID and some other ones. We receive support from them... It’s solely funded by the local government.”

#### Implication of shortage of manpower and lack of staff recruitment

##### Job stress and burn-out

The IDIs revealed how shortage of manpower in the respective PHC centres affected the few health workers working in the PHC centres. The next narration, which describes how the shortage of manpower can affect the existing health workers who are working in the PHC, was by a CNO (head of the facility):

“We have shortage of manpower. Ah! That is number one! Number one! Number one! As you see now, I’m sick. Ah…a...a! There is no way; I can’t rest at home, because there’s only one person on duty this morning and we used to be busy here. I have to manage myself. As you see me now, I’m having serious pain. I used to be here before 8:00 o’ clock every day, but.....today, I couldn’t get up. But when I heard that we have delivery cases (referring to the twin delivery with retained second twin for more than an hour), I have to get up, dress up and come to the clinic.”

##### Prolonged clients’ waiting time

The shortage of health workers was implicated for prolonged waiting time during either antenatal or child welfare clinic visits. Similarly, the observable smaller proportion of nurse-midwives compared with the proportion of other health workers, such as CHOs, CHEWs and HAs, was viewed as unacceptable by all the MOHs and heads of facilities. The MOHs by virtue of their administrative positions, expressed the efforts they had made so far to get the government to rise up to the challenge of staff/patient ratio disproportion in the PHC facilities. For instance, one of the MOHs, while answering questions from the interviewer on his effort so far, in a high-pitch tone, asserted that:

“...it’s not adequate and we have been advocating for the employment of nurses, nurse/midwives to take care of the centres where we run maternity services....”

#### Challenges facing PHC-based maternity centres

The interviews further revealed that the shortage of staff has implication for the operation of the different levels of PHC facilities. Despite the supposed three distinctive levels of PHC facilities to be in operation, each of the facilities in the five LGAs were commonly referred to and inscribed as ‘Primary Health Care Centre.’ An IDI with two of the MOHs revealed that there were three levels of PHC facilities, namely: the health posts/clinics (dispensaries), primary health care centres and comprehensive health centres. They told the interviewer that only the last two (that is, primary health care centres and comprehensive health centres) provide maternity care services. However, each of the facilities studied were inscribed as ‘Primary Health Care Centre’. Thus, no centre was designated as ‘Comprehensive Health Centre’ in all the five local government areas. The other levels of PHC were not included in this study, because maternity services were not being provided there. Below is the explanation credited to one of the medical doctors in an LGA:

“...while the primary health facilities, which are closest to the people at the grassroots, are under the control of the local government. So now, at the primary care level, we have em...some health facilities where we run maternity care service as well and we have some where would just run out-patients’ clinics. That is why at the grassroots level; at the primary level, we have em....different types of health facilities; we have comprehensive health facilities that have primary health care person, then we have health clinics; health posts. So, where you have comprehensive health facilities, you are supposed to have maternity and child health is taken care of. In this local government, at least we have some that fall into that category. Some (not all); at least, we have up to five or six.”

#### Status of staff training and competence

The interviews revealed that the gross shortage of nurse/midwives in the PHC systems led to the situation where CHEWs, HAs and CHOs who were not trained in the art and science of midwifery practice were attending to pregnant women during their respective clinic visits and women in labour. All the IDI participants stressed the fact that the CHEWs and HAs were not trained to attend to women in labour or undertake their child delivery. One of the MOHs confirmed the statement credited to a CNO earlier in this chapter [that is “…The conduct of child delivery is not part of their (CHEWs’ and HAs’) curriculum, they (the CHEWs and the HAs) learn on duty as apprentices” by saying that the nurse-midwives in PHC facilities trained CHEWs and HAs on how to attend to prenatal women and how to undertake child delivery for women in labour in the form of informal ‘master-apprentice’ relationship. His statement is presented below:

“Some of these so called CHEWs and CHOs were initially trained by nurses. And as time goes on, because they have grown in number now, they wanted to take up the jobs of nurses... But those that are knowledgeable about this have been in the forefront of preventing that. They are actually supposed to...to..o..o... (*he stammered*) you know, stay more in the community, whereas the nurses are supposed to be in the maternity centres....”

During the IDI session, it was brought to the notice of one of the MOHs that the CHEWs and HAs used to attempt complicated deliveries, such as multiple pregnancies. On hearing this, the MOH expressed his displeasure with the development in a low-pitch tone thus:

“Well..., ideally, where there are no nurses and midwives, such deliveries (multiple pregnancy delivery) should not even be taken by any other, em...you know…any other health workers. It shouldn’t be! It shouldn’t be! Multiple pregnancies... should be...you know taken care of or the delivery of em...should be handled by a skilled health worker; be it a doctor or a nurse/midwife that has been properly trained”.

The competency of CHEWs, HAs and non-midwife CHOs was observed to be posing challenges to the heads and MOHs in PHC system. From the responses given by the latter category of health professionals, the former group ought to play supportive roles as allied health professionals. During the IDI session involving an MOH, he discussed at length as follows:

“We have, we have the ratio of nurses to the other cadre is so low. Understand? By, by, by, by reason of their training, nurses know how to use partograph, though some of them must have forgotten about it since… it’s not being used as in the teaching hospital. But, some other cadres (the CHEWs and the HAs), they have never been exposed to such a thing. So, you have to train them. You have to supervise them to make sure that they are doing, they are using it well for the purpose for which it’s supposed to be used. But, because for now, there’s no such training... and… they might not get the nitty-gritty of what it’s all about. So, instead of using it to kill more patients, why not, why, why introduce it to them? ...You understand? So, some, so they are sceptical about training them. You know? There’s, there’s lifesaving schemes which ... most nurses have gone through such training. We doctors we have gone through expanded life-saving schemes. We are thinking of modifying the life-saving scheme for the other cadres too. But, that has been, you know, there’s been some..., yes, controversial issues... on it.”

#### Suggestions towards improvement of process

Various suggestions and recommendations that addressed the raised issues under the component process are captured in this session in a concise manner. Some of them are supported with direct quotes from the participants.

#### Staff training

A useful suggestion from the IDIs participants was on training of staff for capacity building. Training of health workers was recommended to be done on a regular basis. One of the MOHs said that he seldom called for resource persons to come to his LGA to train the health workers in PHC facilities. While responding to a question on the issue of training, he argued thus:

“Em… training, you see…, there is a need, you know…, to organize… I do that you know, at my level here. Companies! I will call on them sometimes you say will you sponsor this for us and I will invite you know, resource persons, may be colleagues or senior colleagues from UCH (University College Hospital). Please, will you come? You know em… to give us a lecture on so so area. We use it with projector, I mean with audio visual so we train and there is a need to do this from time to time. For training and retraining because the aspect of training is very, very, crucial when you talk of satisfaction, when you talk of quality of services that is going to be provided; Okay?”

#### Emerging themes from the qualitative strand of the study

The in depth interview generated some addition and supportive themes for the study: These include: shortage of manpower, disproportional health workers/clients ratio, disproportional nurse and midwives/CHEWs and CHOs ratio, lack of framework for staff recruitment, lack of staff training, job stress and burn out.

## Discussion

### Relevance of socio-demographics and manpower strength

The mean age of the health workers who participated in the study was 41 years ±10 SD. More than half of the health workers were within ages 51 to 58 years. In reference to age, the implication of the above is that over 20% of them would have reached the retirement age (which is 65 years) according to the Nigerian civil service rules in the next 14 years [11–13]. Therefore, if no recruitment of health workers takes place within these periods, the number of the manpower needed to provide care in PHC facilities would fall drastically and this may further worsen the reported deplorable state of quality of maternity services at the primary level. Deducing from the statement credited to one of the MOHs who were interviewed, she had never witnessed staff recruitment exercise carried out in the local government where she was serving as MOH for over 15 years. This was the reason another MOH working with a different LGA pleaded for a system of staff recruitment to be established in all LGAs to address the problem of staff leaving service without replacement; a situation that has led to the sub-standard condition reported in this study. Similarly, over one-third of the health workers had worked in the local government facilities for a minimum of 21 years. According to the Nigerian civil service rule, members of staff are retired from civil service when they have worked for 35 years [11–13]. By implication, more than one-third of the workers would have retired in the next 24 years. Again, this presupposes that, if no staff recruitment takes place within this period, more than one-third of the health workers would have left the service without replacement. In addition, there are other routes of exit from service, which are not accounted for in this study. These include death, serious disabilities, migrations, voluntary resignations and termination of appointments for varying reasons.

Most health workers in PHC facilities leave for secondary or tertiary health care and even travel out for greener pastures because of the poor state of the Nigerian PHC. Observably, the PHC in Nigeria lack manpower mostly because the funding is dependent on the government and very poor when compared to secondary and tertiary health care. There is no or weak health insurance with payment mostly out of pocket at the PHC level. So employing more health workers without solving other problems besetting the PHC will not change the situation for better. The focus of our health care should change to strengthening the LGA with emphasis on the PHC. The cumulative effects of these other exit routes could further worsen the nagging human resources shortage in health care system. The health workforce described above had been implicated for disastrous implications for health and well-being of millions of people, including women and their newborns [14]. Thus, the quality of health care services, including the maternity care at PHC, would be grossly compromised as well [15, 16].

In addition, nearly 90% of the health workers were married. This has its own implication for availability of manpower for service provision in the PHC facilities. The health workers were mostly women and since more than three-quarter of them were married not all them would always be available for reproductive health issues such as being away from work on account of maternity leave. This factor has the potential of scaling down the size of the manpower from time to time. In Nigeria, maternity leave has been increased from three to four months in order to promote exclusive breastfeeding among mothers [11–13].

### Status of manpower

Overall, only 26% of the health workers were nurse-midwives. This population of nurse-midwives is too few compared with the client patronage [16, 17]. Other categories of health workers who were not primarily and not formally trained to attend to pregnant women or women in labour but who usually attended to prenatal women and women in labour were relatively far more in number than the nurse-midwives. This disproportionate staff structure could be implicated for the undertaking of complicated child delivery by non-nurses/midwives as observed in one of the PHC facilities during data collection [18]. By extension, this could be one of the reasons for the increased rate of pregnancy-related complications that were usually blamed on PHC facilities by health professionals in other higher levels of health facilities [18]. Since this category of semi-skilled staff offer services that they were not formally trained for to pregnant women, women in labour and their babies, how accurate and reliable are the assessment they are to make on the clients to take prompt decisions and actions? How do they recognise risks in these clients? To find answers to these pertinent questions, this aspect may require further studies. However, their knowledge score in this current study exposed the limit of their skills in midwifery practice. The unskilled may not be able to recognise, manage or refer complications during pregnancy, labour, childbirth and puerperium [19, 20].

### Competence of the health workers

From the findings of the study, it was clear that the majority of the health workers did not demonstrate the level of competence and proficiency expected of professionals trained to undertake antenatal care and child delivery care. More than two-thirds of the health workers had no idea of the concept “Safe Motherhood,” while close to half of the health workers did not know what to do to arrest postpartum bleeding in case it occurred in their facilities. It was confirmed that over 90% of them were attending to pregnant women for clinical assessments and women in labour for childbirth and about 30% of the health workers were not proficient in perineal tear or episiotomy repair. More than 90% were not skilled in Life-Saving Scheme (LSS) and Post Abortion Care (PAC); about 40% could not perform manual removal of retained placenta – a situation that may lead to severe vaginal bleeding. This was because a majority of the health workers were neither nurse-midwives nor midwives and therefore had little or no idea of midwifery practice [21]. All the heads of facilities and MOHs suggested that all the health workers who were neither midwives nor nurse-midwives should go into the community for health promotion and illness prevention programmes. By virtue of their professional training/qualifications, CHEWs are for health outreaches, while the HAs are to provide supportive functions to the nurse/midwives. Again, providing quality intrapartum care and resuscitation is a vital opportunity to ensure a good start in life for all newborns, and skilled birth attendants (midwives) hold the key to quality survival of every new-born [22]. Provision of external source of warmth, including skin-to-skin, enables normal transition from foetal life and reduces deaths from hypothermia and the midwives hold the key as well [23, 24]. Although basic new-born care (BNC) processes are simple, essential and implementable by everyone in every setting where births occur, resuscitation involves relatively more complex processes and the use of bag and mask equipment is generally restricted to skilled, trained birth attendants (nurses, midwives and doctors) [25]. The shortage of midwives compared to other categories of PHC staff is likely to be responsible for ineffective outcome confirmed by the nearly half of health workers who affirmed that the prenatal services provided in their facilities were not effective. Therefore, the disproportional ratio of the nurse-midwives to other categories of PHC staff should be looked into because it is apparent in this study that it contributed to the reported gross deficiencies.

### Non-use of partograph for management of women in labour

In addition, the results of the study confirmed that women in labour were not monitored with partograph (a composite graphical record of maternal and foetal key data during labour; entered against time on a single sheet of paper. Relevant measurements include statistics such as cervical dilation, foetal heart rate, duration of labour and vital signs). Reasons given for non-use were unavailability of partograh and lack of skill to use it. It is expected that a nurse-midwife who is well trained in the use of partograph for monitoring of labour progress could afford to provide a copy by photocopying for each patient he/she is to attend to in labour, especially in situations where conventional patients’ records are recorded inside exercise books owing to unavailability of medical record files. This is capable of causing wrong diagnosis, delayed identification of risk of possible complications and inappropriate intervention [26].

### Nature of services available to women

A situation where over 40% of health workers who attend to women in labour are not trained in LSS and PAC, not skilled enough to perform immediate removal of retained placenta could be detrimental to safe motherhood in the community. The level of proficiency of about 60% of the health workers, who said they could perform the life-saving procedure, irrespective of their qualification, was not evaluated in this study. However, their competence and proficiency are questionable since most of them were neither midwives nor nurse-midwives; this requires further study. This kind of situation, where the population of CHEWS, CHOs and HAs are far more than that of trained nurse-midwives was reported in a study carried out in Ogun State, Nigeria [27].

Through the results of this study, it became obvious that the health workers were not providing some very vital services to mothers and their newborns. Such services include focused antenatal care (FANC) postnatal care and laboratory services (for example, haemoglobin, packed cell volume, neonatal serum bilirubin checks, screening for sexually transmitted infections). Such deprivation puts women and their babies at risk of serious complications during pregnancy, labour and postpartum. For instance, a situation where nearly three-quarter of the health workers confirmed that the serum bilirubin level of neonates born in their facilities was not monitored is not good enough and, therefore, should be discouraged. It is expected that neonatal care in any formal facilities with skilled health personnel, including PHC centres, ought to be better than informal centres in terms of delivering quality care that is evidence-based. However, it is a serious issue when a formal health facility cannot fulfil the expected role. For example, now that discharging healthy term newborns from the hospital after delivery at increasingly earlier postnatal ages has recently become a common practice for medical, social, and economic reasons, it has been shown that newborns whose hospital stay is less than 72 h are at a significantly greater risk for readmission for hyperbilirubinemia (a serious neonatal health problem characterised by jaundice capable of causing severe irreversible brain damage) than those whose stay is 72 h or more [28]. Hyperbilirubinemia is the most commonly reported cause for readmission during the early neonatal period [29].

Moreover, this study reported situations of disrespectful treatments that women received from health workers during antenatal visits or labour. These treatments included denial of choice of position to assume during labour and childbirth, denying women right to choose who to stay with them in labour, enforcement of cooperation on women in labour by bullying, scolding, beating and mishandling. The findings described above were similar to the report of the study done in Sagamu, Nigeria, where approximately two-thirds of the women were unhappy about their involvement in decision-making with respect to birth planning and postpartum contraception [29]. Over half of the respondents also recorded their displeasure on their inability to decide on the providers that attended to them.

In all these respects, the public health care providers are often perceived as less attractive because what women appreciate is rather the personal interest taken in their problems, privacy, continuity of care, and to be treated individually and with respect in friendly surroundings [30]. The health workers’ practice of taking sole decision on whether or not patient’s relations should be allowed into labour room and who should or should not stay with the women in labour is a gross violation of the women’s right [31]. Health workers need education on patients’ rights and choices and should be taught how to involve women in the decision-making process concerning their own maternity care [28]. This study did not identify the reasons for the health workers’ restriction of patients’ relative from labour room; however, it is not out of place if future studies could seek to identify the reasons.

## Conclusion

This study presents the observed shortage of manpower in PHC facilities where maternity services were being provided in general. The evidences of incompetence and delivery maternity-related health care to mothers and their newborns by inappropriate categories of health workers were observed. Poor PHC funding resulting in lack of existing system of recruitments was decried and condemned by heads of facilities. Significant percentage of the health workers were very close to years and/or age of retirements despite no recruitment modalities. Only one medical doctor oversees all PHC facilities in each LGA, despite his/her busy administrative schedule, while there were varying degrees of disproportional ratio of qualified nurse-midwives/CHEWs, HAs. Therefore, the following recommendations are made.

### Recommendations

i. Government should find lasting solutions to the problems of poor funding and movement of PHC health workers, particularly nurse/midwives to greener pastures.
ii. Each LGA should put in place a system of human resources recruitment to ensure regular employment of health workers.
iii. Each LGA should employ more medical officers to work with maternity section in the PHC facilities.
iv. All PHC facilities providing maternity care services should be staffed with adequate number of midwives or nurse-midwives.
v. CHEWs and HAs should be mandated to practice within the limit of their professional training in PHC facilities.

## Abbreviations

CHEWs: Community Health Extension Workers
CHOs: Community Health Officers
CNO: Chief Nursing Officer
FANC: Focused antenatal care
FGD: Focus group discussion
HAs: Health Assistants
IDIs: In-depth Interviews
JCHEWs: Junior Community Health Extension Workers
LGA: Local Government Area
LSS: Life-Saving Scheme
MOH: Medical Officer of Health
PAC: Post-abortions care
PCV: Packed Cell Volume
PHC: Primary Health Care
SDGs: Sustainable Development Goals
STIs: Sexually transmitted Infections
UNICEF: United Nations International Children’s Emergency Fund
USAID: United States Agency for International Development
WHO: World Health Organization

## References

[1] Janssen PA, Saxell L, Page LA, Klein MC, Liston RM, Lee SK (2009). Outcomes of planned home birth with registered midwife versus planned hospital birth with midwife or physician. CMAJ.

[2] Lanre-Abass BA (2008). Poverty and maternal mortality in Nigeria: towards a more viable ethics of modern medical practice. Int J Equity Health.

[3] WHO (2012). Report of the global consultation on producing and developing an appropriate midwifery workforce for low and middle income countries.

[4] NEPAD. Best practice guidelines for implementation of multi-country partnerships intervention model for nursing and midwifery postgraduate education development in Africa. Evaluation Report NEPAD Project*.* 2010*.*

[5] Caughey AB, Cahill AG, Guise JM, Rouse DJ (2014). Safe prevention of the primary cesarean delivery. Am J Obstet Gynecol.

[6] United Nations. Department of Economic. The millennium development goals report 2008. United Nations publications. 2008.

[7] Aluko JO, Oluwatosin A (2008). Pattern and outcome of antenatal care among women attending a catholic mission hospital in Ibadan, Nigeria. Af J Med Med Sci.

[8] Delport CSL, Fourche CB, De Vos AS, Strydom H, Fouche CB, Delport CSL, Van S (2011). Mixed methods research. Research at grass roots for the social sciences and human service professions.

[9] Teddlie C, Tashakkori A. Mixed methods research. The Sage handbook of qualitative research 2011; 285–300.

[10] Strydom H, De Vos AS, Strydom H, Fouche CB, Delport CSL, Van S (2011). Sampling in the quantitative paradigm. Research at grass roots for the social sciences and human service professions.

[11] Nwanolue BOG, Chidubem F (2012). The Nigeriana civil service and promotion of sustainable human development: a critical analysis. Arab J Bus Manag Rev (Oman Chapter).

[12] Briggs BR. Problems of recruitment in civil service: case of the Nigerian civil service. Afr J Bus Manag. 2007;1(6):142–53.

[13] Bayo OA. Federal civil service reform in Nigeria: The case of democratic centralism. J Radix Int Educ Res Consortium. 2012;1(10):1–45.

[14] Crisp N, Gawanas B, Sharp I. Training the health workforce: scaling up, saving lives. The Lancet. 2008;371(9613):689–91. 10.1016/S0140-6736(08)60309-8 18295028

[15] Jeffers JR, Bognanno MF, Bartlett JC (1971). On the demand versus need for medical services and the concept of "shortage". Am J Public Health.

[16] Berk ML, Bernstein AB, Taylor AK. The use and availability of medical care in health manpower shortage areas. Inquiry. 1983:369–80. 6229488

[17] Stanton C (2008). Steps towards achieving skilled attendance at birth. Bull World Health Organ.

[18] Nyango DD, Mutihir JT, Laabes EP, Kigbu JH, Buba M (2014). Skilled attendance: the key challenges to progress in achieving MDG-5 in north Central Nigeria. Afr J Reprod Health.

[19] Adegoke A, Van Den Broek N (2009). Skilled birth attendance-lessons learnt. BJOG Int J Obstet Gynaecol.

[20] Titaley CR, Hunter CL, Dibley MJ, Heywood P (2010). Why do some women still prefer traditional birth attendants and home delivery?: a qualitative study on delivery care services in West Java Province, Indonesia. BMC Pregnancy Childbirth.

[21] Harvey SA, Blandón YCW, McCaw-Binns A, Sandino I, Urbina L, Rodríguez C, Djibrina S (2007). Are skilled birth attendants really skilled? A measurement method, some disturbing results and a potential way forward. Bull World Health Organ.

[22] Blencowe H, Cousens S, Mullany LC, Lee AC, Kerber K, Wall S, Lawn JE (2011). Clean birth and postnatal care practices to reduce neonatal deaths from sepsis and tetanus: a systematic review and delphi estimation of mortality effect. BMC Public Health.

[23] Moore ER, Anderson GC, Bergman N. Early skin-to-skin contact for mothers and their healthy newborn infants. The Cochrane database of systematic reviews. 2007;(3):CD003519-CD003519. 10.1002/14651858.CD003519.pub2 17636727

[24] Kumar V, Shearer J, Kumar A, Darmstadt G (2009). Neonatal hypothermia in low resource settings: a review. J Perinatol.

[25] WHO, UNFPA, & World bank (2012). Maternal mortality in 2005: estimates developed by WHO, UNICEF.

[26] Orhue A, Aziken M, Osemwenkha A (2012). Partograph as a tool for team work management of spontaneous labor. Niger J Clin Pract.

[27] Oladapo OT, Iyaniwura CA, Sule-Odu AO. Quality of antenatal services at the primary care level in southwest Nigeria. Afr J Reprod Health. 2008;12(3):71–92. 19435014

[28] Kaplan M, Hammerman C (2005). Understanding severe hyperbilirubinemia and preventing kernicterus: adjuncts in the interpretation of neonatal serum bilirubin. Clin Chim Acta.

[29] Oladapo OT, Osiberu MO (2009). Do sociodemographic characteristics of pregnant women determine their perception of antenatal care quality?. Matern Child Health J.

[30] Lindmark G, Berendes H, Meirik O (1998). Antenatal care in developed countries. Paediatr Perinat Epidemiol.

[31] Aluko JO, Oyetunde OM (2015). Respectful maternity care: the deserved right of all women. Nursing practice: trends and issues.

